# Breast adenoid cystic carcinoma: An uncommon neoplasm- Case report

**DOI:** 10.1016/j.ijscr.2023.108333

**Published:** 2023-05-17

**Authors:** Mirza Rameez Samar, Wajiha Khan, Mehwish Mooghal, Saba Anjum, Azmina Tajuddin Vali Mohammad, Lubna Mushtaque Vohra

**Affiliations:** a Department of Medical Oncology, Aga Khan University Hospital, Karachi, Pakistan; b Department of Medicine and Surgery, Dow University of Health Sciences, Karachi, Pakistan; c Department of Surgery, Aga Khan University Hospital, Karachi, Pakistan; d Department of Histopathology, Aga Khan University Hospital, Karachi, Pakistan

**Keywords:** Adenoid cystic carcinomas, Triple-negative breast cancer, Early-stage, Breast conservation, Radiotherapy, Case report

## Abstract

**Introduction:**

Adenoid cystic carcinoma is a neoplasm that is commonly of salivary gland origin. It could infrequently arise from other tissues such as breast in which case it behaves favorably despite belonging to triple-negative breast cancer subgroup.

**Case presentation:**

We report a case of a 49-year-old female patient, who presented with right breast pain and upon work-up, was diagnosed with early-stage adenoid cystic carcinoma of the breast. She underwent breast conservation successfully and was advised to get evaluated for adjuvant radiotherapy.

The work has been reported in line with the SCARE criteria (Agha et al., 2020).

**Clinical discussion:**

Breast adenoid cystic carcinoma (BACC) is a rare distinct salivary gland-like carcinoma of the breast with similar morphological features to those seen in salivary gland adenoid cystic carcinoma. Surgical resection is the standard mode of treatment in BACC. The role of adjuvant chemotherapy has not proven beneficial in the management of BACC, owing to the similar survival rates seen in patients with and without chemotherapy.

**Conclusion:**

Localized breast adenoid cystic carcinoma (BACC) is an indolent disease having optimal response to surgical resection alone and thus can omit adjuvant radiotherapy and chemotherapy when completely excised. Our case is unique as BACC is a rare clinical variant of breast cancer with a very low incidence rate.

## Highlights

• Adenoid cystic carcinoma is a neoplasm of salivary gland origin but infrequently arise from other tissues like breast.
• It has a good prognosis, even though it harbors triple negative biology, in part due to its low expression of Ki67 as well as lymph nodal involvement.
• Though uncommon, unilateral painful breast should warrant evaluation for presence of Breast Adenoid Cystic Carcinoma (BACC)

## 1 Background

Breast adenoid cystic carcinoma (BACC) is a unique variety of breast cancer that belongs to triple-negative breast cancer (TNBC) subtype with an annual incidence rate of less than 0.1 % [1]. Most of these cases are known to occur in females aged between 50 and 60 years old with just a few cases reported to date involving males. Morphologically, BACC is a diverse entity that is composed of solid, cribriform and tubular-trabecular pattern found in varying distribution. BACC traditionally presents with a palpable mass with pain in the affected area which according to Kashiwagi et al. occurs as a consequence of perineural infiltration of tumor cells and contraction of myoepithelial cells [2].

BACC is considered a favorable neoplasm that differs from the typical TNBC encountered in medical practice, as evidenced by its lower expression of Ki-67 [3], and rarer involvement of the axillary lymph nodes. Despite these features, it is also found to be notorious for local recurrences and relatively common distant metastasis within 10 years, with the pulmonary disease being the most common site involved [4]. In a study conducted by Zhang, Wenxiang et al. 5-year local recurrence rate was found to be as high as 14.3 % with surgical resection alone [5]. Numerous case series and some population-based studies have been documented, shedding light on the basic management of this breast cancer variant. However, there is still a need for a better understanding of the dynamics of this disease to strategize a tailored treatment plan.

Here, we present a case of a 49 years old premenopausal female, who presented with the chief complaint of pain in the lateral upper and lower quadrants of the right breast, later diagnosed as breast adenoid cystic carcinoma.

The work has been reported in line with the SCARE 2020 criteria: Agha RA, Franchi T, Sohrabi C, Mathew G, for the SCARE Group. The SCARE 2020 Guideline: Updating Consensus Surgical CAse REport (SCARE) Guidelines, International Journal of Surgery 2020;84:226–230 [6].

## 2 Case presentation

A 49 years old, premenopausal female, having a body mass index of 28.8, Para 2 + 0, with no known comorbid, presented in Breast Surgery clinic in July 2022, with a complaint of pain in the lateral upper and lower quadrants of the right breast for 15 days. She had an intra-uterine contraceptive device in place for four years and had no significant family history of malignancy. She had a previous surgical history of cholecystectomy and a right forearm implant after a road traffic accident. Patient had no significant psychosocial history.

**Fig. 1:**
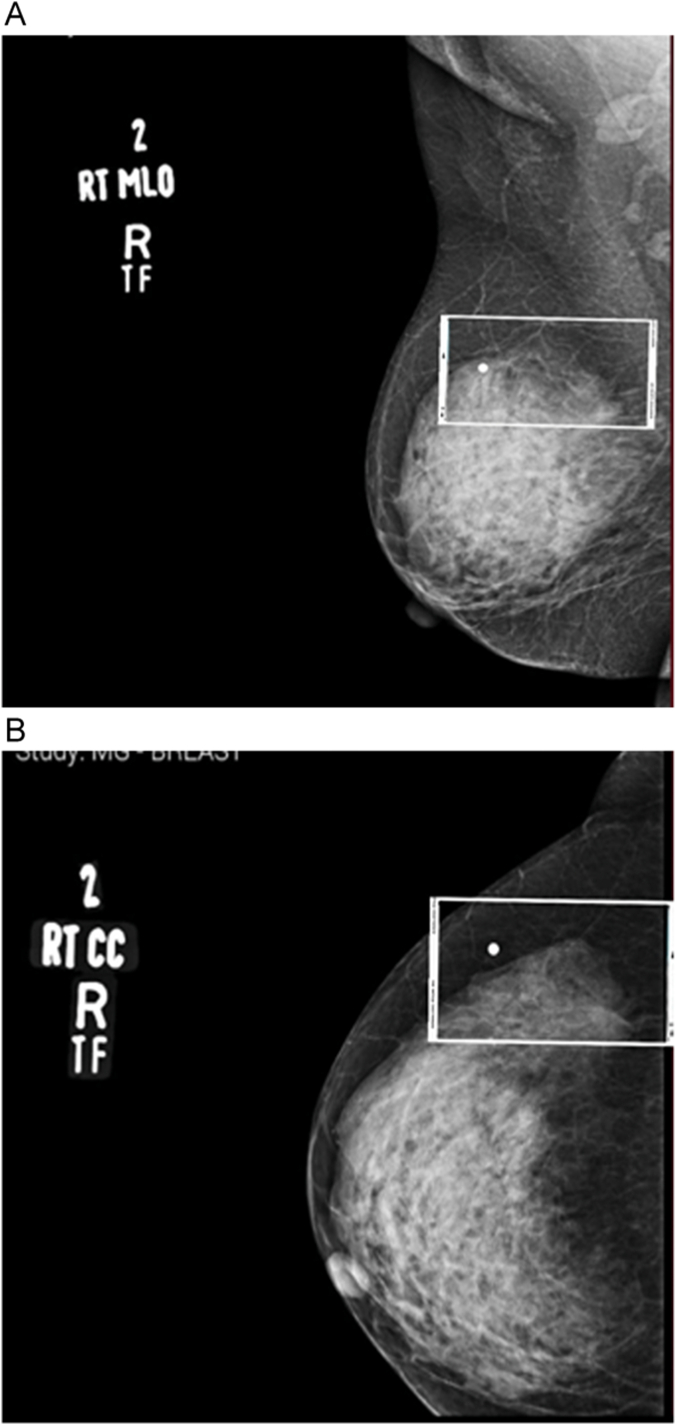
Right breast and axillary MLO (a) and CC (b) view showing asymmetric irregular density in the upper and outer breast.

Her general and physical was unremarkable. Breast examination did not show presence of any underlying lump. Diagnostic mammography was offered which showed an asymmetric irregular density (Fig. 1) in the right upper outer quadrant with no evidence of architectural distortion, skin thickening, or nipple retraction bilaterally (BIRADSCATEGORY-0) but, as heterogeneously dense breast parenchyma was demonstrated bilaterally, an Ultrasound (US) was advised.

**Fig. 2:**
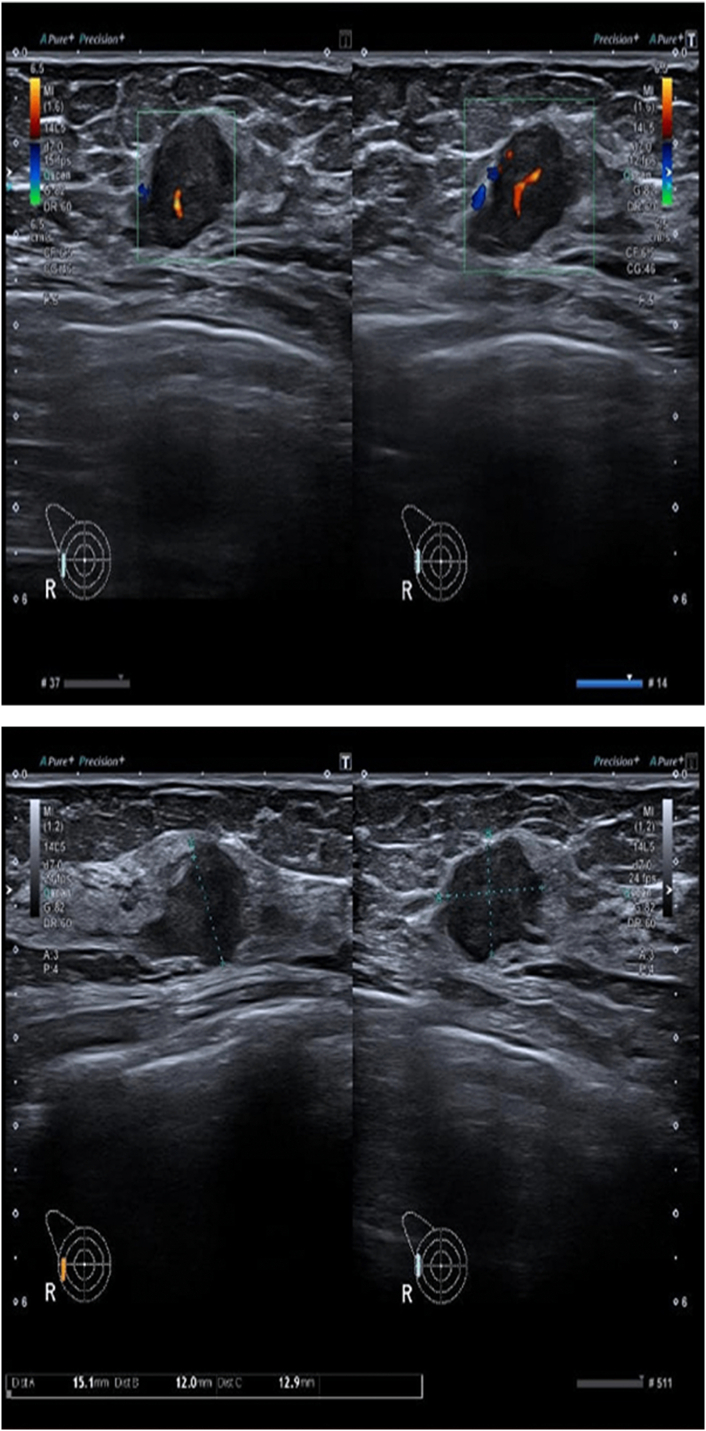
US right breast and axilla showing 15 × 12 × 13 mm hypoechoic solid nodule with ill-defined margins and internal vascularity along with right axillary subcentimetric benign-appearing lymph nodes with intact fatty hila.

On the US of the bilateral breast and axilla (Fig. 2), the right breast had a well-defined hypoechoic solid nodule of 1.5 × 1.2 × 1.3 cm with internal vascularity at 10 o'clock position in correspondence to the site of pain, with the right axilla showing few subcentimetric benign-appearing lymph nodes with intact fatty hila (BIRADS CATEGORY-IV B). The left breast was normal on the US with the left axilla showing few subcentimeter benign-appearing lymph nodes with intact fatty hila (BIRADS CATEGORY-I). Based on the US findings, a right breast biopsy was recommended.

**Fig. 3:**
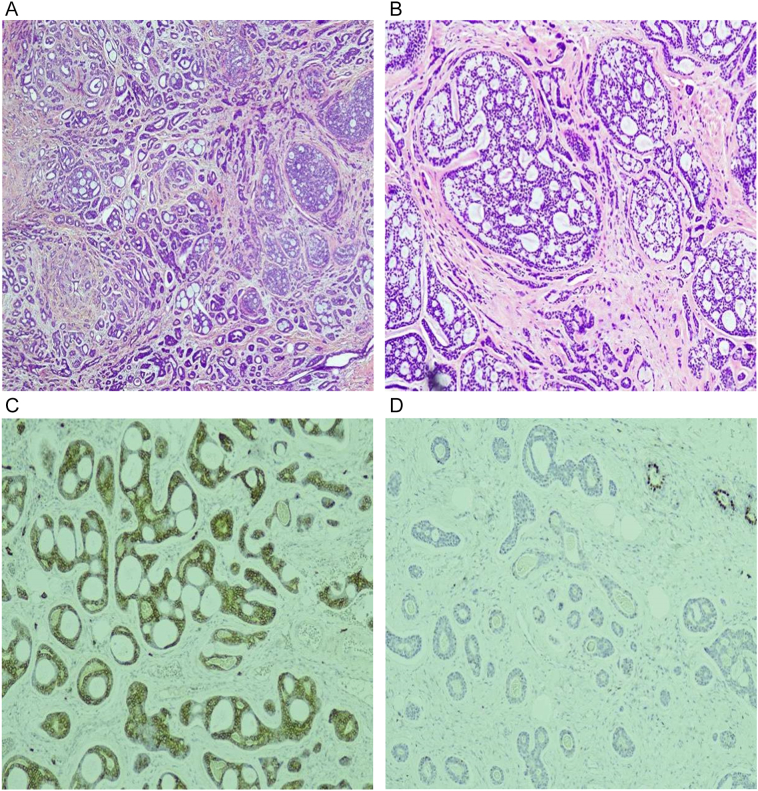
(a) Biphasic tumor comprising ductal and myoepithelial cells in a tubular and cribriform architecture in low power view (b) Biphasic tumor comprising ductal and myoepithelial cells in a tubular and cribriform architecture in high power view (c) IHC staining of ER being negative in tumor and positive in residual benign breast ducts (d) IHC staining of CD117 being positive in tumor and negative in residual benign breast ducts.

Core biopsy of right breast 9 o'clock lesion showed a biphasic tumor predominantly of cribriform architecture consisting of ductal and myoepithelial cells (containing myxoid and hyalinized globules). Immunohistochemical (IHC) staining of the specimen highlighted p63 and CD117 whereas ER (Estrogen Receptor), as well as GATA3, came out to be negative. These features were consistent with adenoid cystic carcinoma. Metastatic workup including computed tomography (CT) chest, whole abdomen, pelvis, and whole-body bone scintigraphy, turned out to be negative making the clinical stage of T1N0M0. The patient subsequently underwent right breast wire localized wide local excision (WLE) and sentinel lymph node biopsy (SLNBx). The procedure was conducted by the highly skilled surgeon of our tertiary care hospital with a working experience of 10 years (she has done MBBS, FCPS general surgery, FCPS breast surgery, FACS). The histopathological report revealed a tumor size of 2.1 × 1.6 × 1.3 cm, unifocal with margins and skin being tumor free, without any evidence of ductal carcinoma in situ (DCIS) or lobular carcinoma in situ (LCIS). Five sentinel lymph nodes were taken out, none of which was involved by the tumor. Thus, it was pathologically staged II-A i.e. pT2N0M0 (Fig. 3).

She recovered well, her case was discussed in multi-disciplinary tumor board. She was referred to Radiation Oncology for adjuvant radiotherapy on account of breast conservation. Subsequently, she received 26 Fractions of radiation to right breast, comprising of 36 Gy (Gy). She has since then been kept on surveillance and on subsequent three and six months follow up patient is doing well, with satisfactory local and systemic examination findings.

## 3 Discussion

Breast adenoid cystic carcinoma (BACC) is a rare distinct salivary gland-like carcinoma of the breast with similar morphological features to those seen in salivary gland adenoid cystic carcinoma i.e. intermixed gland-forming luminal cells with myoepithelial or basal cells, resulting in a pseudolumen that contains abundant basement membrane material. It was first known as ‘Cylindroma’ in the early 1850s by Billroth. The name was later changed to adenoid cystic carcinoma in 1945 by Geschikter [7]. In literature, BACC has been reported to occur mostly as a solitary mass in the superior lateral quadrant or below the areola of the breast, with only rare presentations with multiple masses [8].

BACC is often found to have a basal-like phenotype with negative immunohistochemical stain for estrogen receptor (ER), progesterone receptor (PR), and human epidermal growth factor receptor 2 (HER-2). Inspite of being triple negative, it presents and behaves differently with fewer distant metastasis, nodal involvement, and local recurrences when completely excised. Histologically various patterns (cribriform, tubular, or solid) can be appreciated. Among which, cribriform is the commonest pattern whereas solid is the most aggressive pattern, especially the basaloid variant [9].

Surgical resection is the standard mode of treatment in BACC. In an institutional study of 20 years duration, BACC accounted for 0.1 % of all breast cancer diagnosed, with most patients presenting with stage I-II disease, which were successfully treated with surgical resection alone [7].

Adjuvant radiotherapy is usually indicated in cases of nodal metastases. It is also recommended following breast conservation due to the higher rate of positive surgical margins which in turn increases the risk of local recurrences. In a study conducted by Sun, Jia-Yuan et al., adjuvant radiotherapy after lumpectomy increased the 5-year cancer-specific survival by 4.3 % [10]. In another study, post-operative radiation was associated with better survival rates than without radiation [11]. The role of adjuvant chemotherapy has not proven beneficial in the management of BACC, owing to the similar survival rates seen in patients with and without chemotherapy [12]. In a study involving 19,900 patients, the survival rates of BACC were found to be statistically similar to infiltrating ductal carcinomas [13]. Thus, the common indications for adjuvant chemotherapy, recommended by the present-day guidelines for the diagnosis and treatment of TNBC, cannot be implied upon BACC.

As this disease is rare in its occurrence, specific guidelines regarding the optimal diagnosis and management of BACC have not been made. Therefore, more studies are required to reach a consensus to guide the tailoring of an organized treatment plan for such breast cancer variants.

## 4 Conclusion

Breast adenoid cystic carcinoma is a distinct variant in breast cancer, which despite being included among the heterogeneous triple negative histology, has a good prognosis and responds well to surgical resection, with mastectomy alone providing acceptable clinical outcomes.

## Abbreviations

BACC: Breast adenoid cystic carcinoma
TNBC: Triple-negative breast cancer
US: Ultrasound
IHC: Immunohistochemical staining
CT: Computed tomography
WLE: Wide local excision
SLNBx: Sentinel lymph node biopsy
DCIS: Ductal carcinoma in situ
LCIS: Lobular carcinoma in situ
ER: Estrogen receptor
PR: Progesterone receptor
HER-2: Human epidermal growth factor receptor 2

## Consent to participate

Not applicable.

## Consent for publication

Written informed consent was obtained from the patient for publication of this case report and any accompanying images. A copy of the written consent is available for review by the Editor-in-Chief of this journal.

## CRediT authorship contribution statement

1. Guarantor of integrity of the entire study: Dr. Mirza Rameez Samar, Dr. Wajiha Khan, Dr. Mehwish Mooghal, Dr. Saba Anjum, Dr. Azmina Tajuddin Vali Mohammad, Dr. Lubna Vohra.

2. Study concepts and design: Dr. Mirza Rameez Samar, Dr. Wajiha Khan, Dr. Mehwish Mooghal, Dr. Lubna Vohra.

3. Literature research: Dr. Mirza Rameez Samar, Dr. Wajiha Khan, Dr. Mehwish Mooghal.

4. Manuscript preparation: Dr. Mirza Rameez Samar, Dr. Wajiha Khan, Dr. Mehwish Mooghal, Dr. Lubna Vohra.

5. Manuscript editing: Dr. Mirza Rameez Samar, Dr. Wajiha Khan, Dr. Mehwish Mooghal, Dr. Lubna Vohra.

## Funding

Not applicable.

## Provenance and peer review

Not commissioned, externally peer reviewed.

## Ethical approval

Not applicable as case reports are exempted from the provision of ethical approval in our institute.

## Research registration

N/A.

## Guarantor

Dr. Mirza Rameez Samar, Dr. Wajiha Khan, Dr. Mehwish Mooghal, Dr. Saba Anjum, Dr. Azmina Tajuddin Vali Mohammad, Dr. Lubna Vohra.

## Declaration of competing interest

The authors declare that they have no competing interests.
